# Functional Characterization of the Pheophytinase Gene, *ZjPPH*, From *Zoysia japonica* in Regulating Chlorophyll Degradation and Photosynthesis

**DOI:** 10.3389/fpls.2021.786570

**Published:** 2021-12-23

**Authors:** Ke Teng, Yuesen Yue, Hui Zhang, Hui Li, Lixin Xu, Chao Han, Xifeng Fan, Juying Wu

**Affiliations:** ^1^Beijing Academy of Agriculture and Forestry Sciences, Beijing, China; ^2^College of Grassland Science, Beijing Forestry University, Beijing, China

**Keywords:** pheophytinase, chlorophyll degradation, photosynthesis, chlorophyll *a* fluorescence, senescence, *Zoysia japonica*

## Abstract

Pheophytinase (PPH), the phytol hydrolase, plays important roles in chlorophyll degradation. Nevertheless, little attention has been paid to the PPHs in warm-season grass species; neither its detailed function in photosynthesis has been systematically explored to date. In this study, we isolated *ZjPPH* from *Zoysia japonica*, an excellent warm-season turfgrass species. Quantitative real-time PCR analysis and promoter activity characterization revealed that the expression of *ZjPPH* could be induced by senescence, ABA, and dark induction. Subcellular localization observation proved that ZjPPH was localized in the chloroplasts. Overexpression of *ZjPPH* accelerated the chlorophyll degradation and rescued the stay-green phenotype of the Arabidopsis *pph* mutant. Moreover, *ZjPPH* promoted senescence with the accumulation of ABA and soluble sugar contents, as well as the increased transcriptional level of *SAG12* and *SAG14*. Transmission electron microscopy investigation revealed that *ZjPPH* caused the decomposition of chloroplasts ultrastructure in stable transformed Arabidopsis. Furthermore, chlorophyll *a* fluorescence transient measurement analysis suggested that *ZjPPH* suppressed photosynthesis efficiency by mainly suppressing both photosystem II (PSII) and photosystem I (PSI). In conclusion, *ZjPPH* plays an important role in chlorophyll degradation and senescence. It could be a valuable target for genetic editing to cultivate new germplasms with stay-green performance and improved photosynthetic efficiency.

## Introduction

Chlorophyll degradation is the phenotype landmark of leaf senescence processes in plants. The biochemistry and topology of chlorophyll degradation have been explored systematically during the recent decades (Hortensteiner, 2013; Kuai et al., 2018). Specifically, the identification of important *stay-green* mutants unmasked the key catalyzing steps in the chlorophyll breakdown pathway. In brief, the breakdown of chlorophyll was started with the conversion of chlorophyll *b* (Chl *b*) to Chl *a* catalyzed by two Chl *b* reductase paralogous, NYC1 and NOL (Yu et al., 2021). Next, magnesium was released from Chl *a* by the STAY-GREEN protein (SGR) to yield pheophytin *a* (Shimoda et al., 2016). Then, the pheophytin *a* was converted to pheophorbide *a* with the action of pheophytinase (PPH) (Zhang et al., 2016). Finally, the pheophorbide *a* was subsequently catalyzed by PAO, RCCR, and TIC55, generating red chlorophyll catabolite (RCC), phyllobilin (*p*FCC), and hydroxy-*p*FCC, respectively (Kuai et al., 2018). The above steps were finished inside the chloroplasts and were termed as the first part of the chlorophyll degradation pathway. The other part of the pathway resulted in a diversity of phyllobilins and took place outside the chloroplasts (Guo et al., 2021).

The *PPH* gene was first reported to be involved in chlorophyll breakdown machinery during leaf senescence in Arabidopsis (Schelbert et al., 2009) and rice (Morita et al., 2009). Bamboo (*Bambusa emeiensis*) PPH (also called BeCRN1) is localized in the chloroplasts, and its constitutive overexpression rescued the stay-green phenotype of Arabidopsis *crn1* mutants (Wei et al., 2013). Knockdown of *SlPPH* delayed chlorophyll breakdown and accumulated pheophytin during leaf senescence in tomato (*Solanum lycopersicum*) (Guyer et al., 2014). Perennial ryegrass (*Lolium perenne*) *PPH* was reported to accelerate chlorophyll degradation and might be a direct downstream target gene of abscisic acid and cytokinin-related transcription factors (Zhang et al., 2016). Apple (*Malus domestica*) *MdPPH*, a target of *MdERF17*, was required for peel Chl degradation and pigment accumulation (Han et al., 2018). Warm-season grasses are important for both agricultural production and ecosystem services (Lee et al., 2019). Nevertheless, little attention has been paid to the *PPH* gene in warm-season grass species; neither its function has been explored to date.

The popularization of Chl *a* fluorescence transient measurements (JIP test) provides us a convenient and reliable tool to evaluate the status and performance of photosystem II (PSII) reaction centers, photosynthetic electron transport, and the attribute of both the donor and acceptor sides of PSII (Hu et al., 2016; Kalaji et al., 2016, 2018; Zagorchev et al., 2021). JIP test revealed that the overexpression of the γ-tocopherol methyl transferase gene improved the efficiency of photosystem and consequently enhanced the drought adaptability of transgenic *Brassica juncea* plants (Yusuf et al., 2010). Analysis of Chl *a* fluorescence indicated that the heterologous expression of wheat *TaFBA1* alleviated the damage to the photosynthetic apparatus of transgenic tobacco under high salinity (Zhao et al., 2017). JIP test demonstrated that the overexpression of *Fld* preserved the integrity and functionality of the photosynthetic machinery in tobacco leaves infected with *Botrytis cinereal* (Rossi et al., 2017). However, the detailed influence of *PPH*, the catalyzer of the primary electron acceptor in PSII (pheophytin *a*), on photosystem remains poorly explored. The question on the specific functional machinery of *PPH* in photosynthesis still has unfilled gaps.

*Zoysia* grass is an excellent warm-season turfgrass species and is widely utilized in football pitches and landscaping (Xuan et al., 2013; Chen et al., 2015). Compared with the cool-season turfgrasses, the most limiting factor preventing the popularization of *Zoysia japonica* is the shorter green period (Teng et al., 2016). Consequently, getting a better comprehension of the chlorophyll degradation and photosynthetic mechanisms is important. The objectives of this study were to characterize *ZjPPH* in controlling chlorophyll degradation and to investigate its detailed regulatory mechanism in photosynthesis. It will contribute to breeding new *Z. japonica* cultivar with improved quality in the future.

## Materials and Methods

### Plant Materials and Growth Conditions

*Zoysia japonica* cultivar “Zenith” seeds, purchased from the Patten Seed Company (Lakeland, GA, United States), were cultivated in the TS1 nutrient medium (Klasmann-Deilmann, Germany). The seedlings were kept in an RXZ-380D-LED growth chamber (Ningbo Jiang Nan Instrument Factory, Ningbo, China), which is set at 28/25^ o^C (day/night), 65% humidity, and approximately 400 μmol m^–2^s^–1^photosynthetic active radiation (PAR). *Arabidopsis thaliana* “Columbia” (wild-type background) and *pph* mutant (SALK_000095) were kept in a growth chamber set at 24/22^ o^C (day/night) with 65% humidity and 400 μmol m^–2^s^–1^ PAR. The photoperiod of the growth chambers was set to 16 h per 24 h. *Nicotiana benthamiana* was cultivated under identical growth conditions as *Z. japonica.* The plants were irrigated by 1/2 strength Hoagland’s solution weekly (Hoagland and Arnon, 1950).

### Cloning of *ZjPPH* and Its Promoter

Total RNA was extracted from “Zenith” leaves using Trizol and then it was used to generate cDNA using PrimeScript RT reagent kit (TaKaRa, Dalian, China). Genomic DNA was extracted using the CTAB methods from fresh leaves of Zenith. BLAST analysis was carried out using homologous proteins as queries in the *Zoysia* grass genome sequence to design primers used for cloning *ZjPPH* (Table 1). PCR amplification was performed to amplify the *ZjPPH* gene and its promoter sequence, respectively. The PCR products were then purified and inserted into the pMD-19T cloning vector (TaKaRa, Dalian, China) with ampicillin resistance. After antibiotic selection and PCR verification, the plasmids containing the complete coding sequence (CDS) (pMD-ZjPPH) and the promoter sequence (pMD-ZjPPHpro) from the base 0 were generated, respectively. The correct plasmids examined by Sanger sequencing and alignment were selected and used in the subsequent experiments.

**TABLE 1:** Primers used for gene cloning, expression analysis, and plasmids construction.

Primer names	Primer sequence (5′-3′)
ZjPPH-F	AGAGTTAGTGCGTCAGCCACC
ZjPPH-R	CTGCTGTAAGGACAAGTTATCTGGA
ZjPPHpro-F	ATTGTTCGGGCTAATGGTGTTC
ZjPPHpro-R	CACATCACCCACATAGAATCCC
qZjPPH-F	ATAAGCAGGTTCAGCATTTCGT
qZjPPH-R	CCATGACCCAGACTAAGACCACTA
qZjACT-F	GGTCCTCTTCCAGCCATCCTTC
qZjACT-R	GTGCAAGGGCAGTGATCTCCTTG
qGUS-F	GCTCTACACCACGCCGAACA
qGUS-R	GTCCCGCTAGTGCCTTGTCC
Y3-ZjPPH-F	cctactagtcctagggacgtcaATGGAAGTGGTGTCTTGCAG
Y3-ZjPPH-R	tgctcaccatacgcgttacagaTCTGGAAACTACCCGTGAGT
pTA7002-ZjPPH-F	ggacacgctgaagctagtcgacATGGAAGTGGTGTCTTGCAG
pTA7002-ZjPPH-R	gggaggcctggatcgactagtcTTATCTGGAAACTACCCGTG
1391-ZjPPH-Pro-F	aagcctagggaggagtccacATTGTTCGGGCTAATGGTGTTC
1391-ZjPPH-Pro-R	tttaccctcagatctaccatAATCGGCTACGGTGGCTGAC
qAtSAG12-F	ACAACGTCGAACGCATTGAACAT
qAtSAG12-R	TGCCGAGACACCTTTGAAACCAG
qAtSAG14-F	ATGTGGCAGTTGTATCAGAAGC
qAtSAG12-R	GGTGTTTAGCATAATTTTGACCGGA
qAtCAB1-F	AGGAACCGTGAACTAGAAGTTATC
qAtCAB1-R	CCGAACTTGACTCCGTTTCT
qAtPsaF-F	ACGGGAAGTACGGATTGTTATG
qAtPsaF-R	CGATCCATCCAGCAATGTAGAG
qAtRbcL-F	GGGTTCAAAGCTGGTGTTAAAG
qAtRbcL-R	CTCGGAATGCTGCCAAGATA
qAtRCA-F	GTCCAACTTGCCGAGACCTAC
qAtRCA-R	TTTACTTGCTGGGCTCCTTTT
qAtUBQ-F	AGTCCACCCTTCATCTTGTTCTC
qAtUBQ-R	GTCAGCCAAAGTTCTTCCATCT

### Quantitative Real-Time PCR

Total RNA was isolated from different organs of *Z. japonica*, including roots, stems, and leaves, separately. Total RNA samples were also extracted from leaves at different senescent stages as described previously (Teng et al., 2016). Phytohormone treatments, including 10 μmol ABA, 10 μmol MeJA, and 0.5 mM SA, and dark induction were applied to investigate the expression characteristics following a protocol described by Teng et al. (2017). qRT-PCR data calculation was performed using the ΔΔCt method (Livak and Schmittgen, 2001). The *Z. japonica* β*-actin* gene (GenBank accession No. GU290546) was used as the housekeeping gene (Teng et al., 2017).

### Bioinformatic Analysis of *ZjPPH* and Its Promoter Sequence

The NCBI BLAST analysis was carried out to search for the homologs. Phylogenetic tree was built with the neighbor-joining method using MEGA v.5 software (Tamura et al., 2011). The Ks/Ka ratio was generated using DnaSP6 software (Rozas et al., 2017). Based on the PlantCARE online database, the *cis*-elements in the promoter sequence were identified (Lescot et al., 2002). The molecular weight (MW) and the theoretical isoelectric point (PI) were calculated using the compute pI/Mw tool^1^. The subcellular localization pattern was predicted using the online program TargetP 1^2^.

### Plasmid Construction

The *ZjPPH* CDS was inserted into the plant binary vector 3302Y3 (Jia et al., 2016) to generate the 35S:ZjPPH:YFP construct used for subcellular localization investigation. The *ZjPPH* CDS was infused into the pTA7002 vector used for generating transgenic Arabidopsis lines. The promoter sequence of *ZjPPH* was inserted into the pCambia1391Z vector to generate the ZjPPHpro:GUS construct used for the GUS staining determination.

### Subcellular Localization Observation

For the subcellular localization observation, *Agrobacterium tumefaciens* EHA105 harboring 35S:ZjPPH:YFP was transformed into the lower epidermal cells of *N. benthamiana* leaves using an injection syringe according to the transient expression protocol (Sparkes et al., 2006). After 48-h incubation, the fluorescence in the tobacco cells were checked and photographed using a SP8 laser confocal scanning microscope (Leica, Mannheim, Germany).

### Generation of Transgenic *Arabidopsis thaliana* Lines and Physiological Determinations

The *A. tumefaciens* GV3101 containing the pTA7002-ZjPPH construct, and pTA7002 empty vector were used to generate transgenic and control *A. thaliana* lines using the floral dip method, respectively (Clough and Bent, 1998). The seeds harvested were screened on MS medium supplemented with 30 mg L^–1^ hygromycin. After PCR verification, T_3_ lines with 100% hygromycin resistance were selected for further morphological assessment. Three-week-old healthy lines growing in MS medium were transplanted to filter paper immersed in 30 μM dexamethasone (DEX) (Sigma-Aldrich, Munich, Germany) and 0.01% Tween-20 solution diluted with ddH_2_O. After 4 days of induction, the plants were photographed in a photostudio using an EOS 80D digital camera (Canon, Tokyo, Japan).

The chlorophyll content was extracted using 95% ethanol, and total chlorophyll contents were then quantified based on the absorbance recorded (Teng et al., 2016). ABA content was measured using an ELISA kit (H251, Jiancheng Bioengineering Institute, Nanjing, China) based on the protocol provided by the manufacturer. Soluble sugar content was determined using the 3,5-dinitrosalicylic acid method (Sun et al., 2020). Leaf fresh weight was adopted in assessing these indicators above.

A Handy PEA analyzer (Hansatech, Kings Lynn, United Kingdom) was used for the determination of chlorophyll fluorescence parameters. For dark incubation, fresh leaves were covered by a leaf clip at the middle of the leaf blades for 30 min. Then, the clips were removed and chlorophyll fluorescence was immediately measured by exposing the leaves to a two-second saturating light pulse of 3,500 μmol photons m^–2^ s^–1^. Photosynthetic parameters were recorded and calculated according to the protocol provided by the manufacture. Ten replicates were utilized for each of the CK, line-3, and line-7. The parameters were processed using Handy PEA v.1.3 software. Origin Pro v.2019b (OriginLab Corporation, Northampton, MA, United States) was used for data presentation.

### Transmission Electron Microscopy Observation

Transmission electron microscopy observation was performed based on the protocol of Park et al. (2007) with moderate modifications. After DEX incubation, leaf tissues were soaked in the fixative buffer with 2% paraformaldehyde, 2% glutaraldehyde, and 50 mM sodium cacodylate (pH 7.2). Next, the samples were washed with 50 mM sodium cacodylate buffer (pH 7.2) three times at 4^°^C. Then, the samples were treated in the 50 mM sodium cacodylate buffer containing 1% osmium tetroxide (pH 7.2) at 4^°^C for 2 h. After being washed three times with distilled water at 25^°^C, the samples were incubated in 0.5% uranyl acetate for at least 30 min at 4^°^C. A gradient series of propylene oxide and ethanol was used for sample dehydration. Finally, the samples were embedded in resin for generating ultrathin sections using an ultra-microtome. The samples were then placed on copper grids after being polymerized at 70^°^C for 24 h. Finally, they were treated with 2% uranyl acetate for 5 min and with Reynolds’ lead citrate for 2 min at 25^°^C. The ultrastructure of chloroplasts was then observed and photographed using a HT7700 transmission electron microscope (Hitachi, Tokyo, Japan). We examined 10 cells in each sample and photographed the representative chloroplast structure in well-cut sections.

### Statistical Analysis

Data were analyzed through Student’s *t*-test or one-way ANOVA using SPSS version 15.0 (IBM, Chicago, IL, United States). *p < 0.05 were recognized statistically significant. All data are presented as the mean ± SD (*n* = 3 at least).

## Results

### Cloning and Bioinformatic Analysis of *ZjPPH*

**FIGURE 1:**
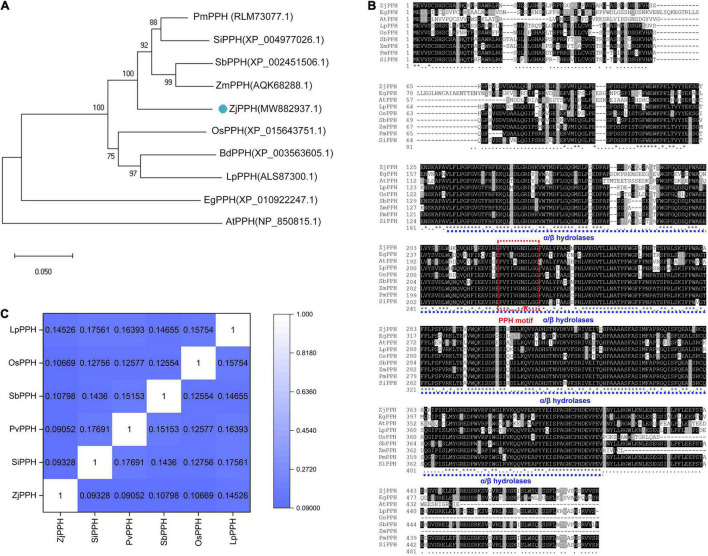
Bioinformatic analysis of *ZjPPH*. **(A)** Phylogenetic tree: orthologous proteins in *Panicum miliaceum* (PmPPH), *Setaria italica* (SiPPH), *Sorghum bicolor* (SbPPH), *Zea mays* (ZmPPH), *Zoysia japonica* (ZjPPH), *Oryza sativa* (OsPPH), *Brachypodium distachyon* (BdPPH), *Lolium perenne* (LpPPH), *Elaeis guineensis* (EgPPH), and *Arabidopsis thaliana* (AtPPH) were used for analysis. **(B)** Multiple sequences alignment and conserved domain prediction. **(C)** Ka/Ks values.

The *ZjPPH* (GenBank accession No.:MW882937) was successfully cloned by RT-PCR based on the genome sequence database of *Z. japonica*. The coding sequence of *ZjPPH* is 1479 bp in length, encoding 492 amino acids. Its theoretical pI and MW are 8.19 and 54.67 kD, respectively. A phylogenetic tree was constructed and indicated that ZjPPH was most closely related to the PPH orthologs in *Zea mays* and *Sorghum bicolor* (Figure 1A). Conserved domain analysis revealed that ZjPPH contained a conserved domain of α/β hydrolases and a PPH motif (PVYIVGNSLGG) containing a catalytic Ser residue (Figure 1B). The synonymous and non-synonymous substitution rates (Ka/Ks) were calculated to classify the evolutionary selection types. It showed that the Ka/Ks values of ZjPPH were less than one, suggesting that ZjPPH is under purifying selection (Figure 1C).

### Isolation of Promoter and GUS Staining Assay

**FIGURE 2:**
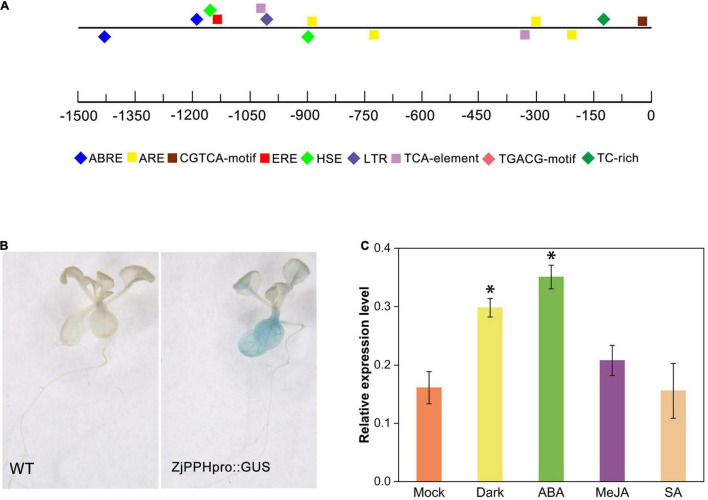
Promoter analysis and GUS assay. **(A)** cis-elements prediction; **(B)** GUS staining results. **(C)** *GUS* expression level under different treatments. *Indicate significant differences at *p* ≤ 0.05 based on Student’s *t*-test.

A 1500-bp nucleotide promoter sequence was obtained by PCR amplification. *Cis*-elements prediction results demonstrated that the *ZjPPH* promoter contained two ABRE elements involved in ABA responsiveness, one CGTCA motif and one TGACG motif involved in MeJA-responsiveness, two TCA elements participant in salicylic acid responsiveness, and one ERE elements correlated with ethylene-responsive element (Figure 2A).

Using transgenic Arabidopsis seedlings, GUS histochemical staining was performed to further characterize the promoter. It displayed that the promoter sequence could drive GUS expression in transgenic plants. Specifically, blue pattern was abundantly found in the leaves and root (Figure 2B). The *GUS* expression level showed that the 48 h of dark and ABA treatment could induce the expression of *GUS* gene, whereas the MeJA and SA treatment caused no significant changes, indicating the role of ABA and dark treatment in regulating the promoter activity (Figure 2C).

### Transcriptional Expression Characteristics of *ZjPPH*

**FIGURE 3:**
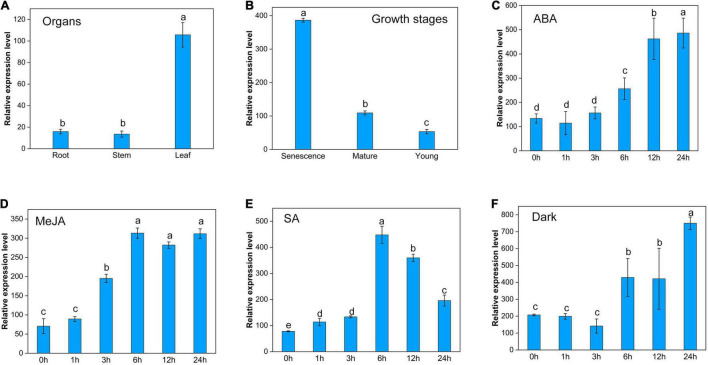
Transcriptional expression characters of ZjPPH. **(A)** Different organs. **(B)** Leaves at different growth stages. Continuous expression tendency under different treatments: ABA **(C)**, MeJA **(D)**, SA **(E)**, and dark **(F)**. Data were expressed as mean ± SD (*n* = 4). Different letters indicate significant differences at *p* ≤ 0.05 based on Fisher’s protected least significant difference (LSD) test.

The expression of *ZjPPH* exhibited a tissue-specific character with a higher level in the leaf than in the root and stem (Figure 3A). The expression level of *ZjPPH* was found more abundantly in senescent leaves than in mature and young leaves (Figure 3B). Hormone inducement analysis revealed that the transcriptional activity of *ZjPPH* could be activated by ABA, MeJA, SA, and dark treatment within 24 h of treatment (Figures 3C–F). Specifically, ABA and dark treatments upregulated the expression level of *ZjPPH* from 6 h and maintained a higher level at 24 h. MeJA treatment induced the expression of *ZjPPH* from 3 h and continuously kept the highest level from 6 h to the end of the experiment. SA increased the expression of *ZjPPH* from 1 h and reached the highest level at 6 h, and then it decreased gradually from 6 to 24 h.

### Subcellular Localization of *ZjPPH*

**FIGURE 4:**
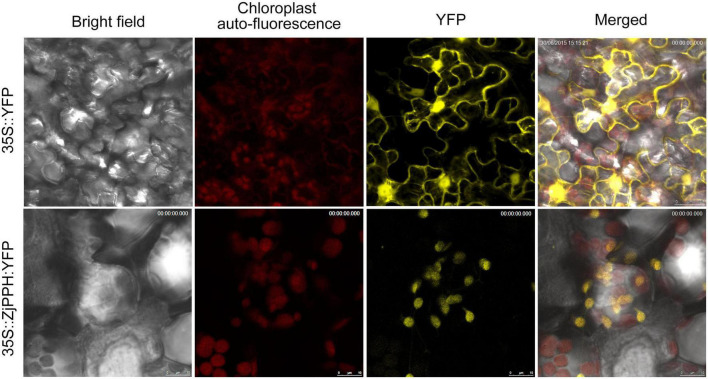
Subcellular localization of ZjPPH.

The TargetP 1.1 online program demonstrated that the ZjPPH was a chloroplast-specific localized protein. To further verify the subcellular localization pattern of ZjPPH, transient expression analysis was employed. As shown in Figure 4, the tobacco lower epidermis cells transformed with 35S:YFP were full of YFP signal. In contrast, the YFP signal was only detected in the chloroplast of the tobacco cells transformed with 35S:ZjPPH:YFP construct. The results proved that ZjPPH was a chloroplast-specific localized protein.

### Overexpression of *ZjPPH* Accelerated Chlorophyll Degradation and Senescence

**FIGURE 5:**
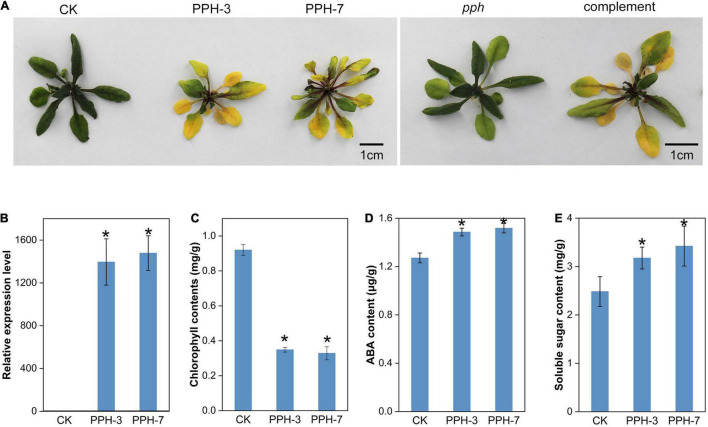
Phenotype observation and physiological determination of *ZjPPH*-overexpressing lines. **(A)** Picture of *ZjPPH*-overexpressing lines and complementary line. **(B)** Expression-level assessment of *ZjPPH* in transgenic lines. **(C)** Chlorophyll contents determination. **(D)** ABA content. **(E)** Soluble sugar content. Data were expressed as mean ± SD (*n* = 4). *Indicate significant differences at *p* ≤ 0.05 based on Student’s *t*-test.

Transgenic Arabidopsis lines overexpressing *ZjPPH* were generated to explore the function of *ZjPPH*. The *ZjPPH*-overexpressing Arabidopsis lines showed an obvious yellowing phenotype compared with control (Figure 5A). The qRT-PCR analysis proved that the *ZjPPH* was efficiently expressed in the transgenic lines (Figure 5B). Correspondingly, chlorophyll content determination results showed that the chlorophyll contents was higher in the control than in the transgenics (Figure 5C). Moreover, *ZjPPH* also rescued the stay-green phenotype of the *pph* mutant (Figure 5A). ABA content determination showed that overexpression of *ZjPPH* increased the ABA content in transgenic lines compared with control (Figure 5D). The soluble sugar content was also found to be higher in the transgenic lines (Figure 5E).

**FIGURE 6:**
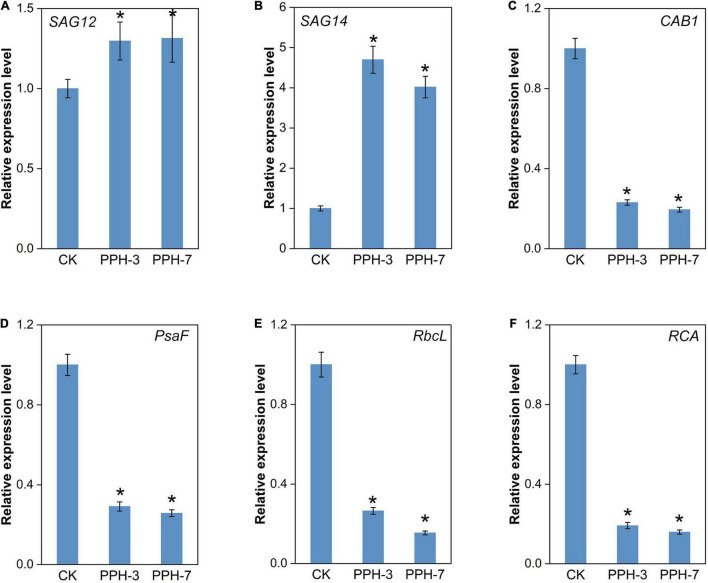
Expression analysis of senescence markers and photosynthesis-related genes. **(A)** *SAG12*. **(B)** *SAG14*. **(C)** *CAB1*. **(D)** *PsaF*. **(E)** *RbcL*. **(F)** *RCA*. Data were expressed as mean ± SD (*n* = 4). *Indicate significant differences at *p* ≤ 0.05 based on Student’s *t*-test.

Two senescence marker genes, *SAG12* and *SAG14*, were selected to assess the impact of *ZjPPH* in senescence processes. It turned out that the expression levels of the two senescence marker genes were upregulated significantly (Figures 6A,B). It indicated that the overexpression of *ZjPPH* promoted senescence. In addition, the expression of four photosynthetic efficiency marker genes was monitored to evaluate the photosynthetic rate. The results revealed that the overexpression of *ZjPPH* significantly downregulated the expression of *CAB1* (Chl a/b binding protein 1), *PsaF* (PSI component), *RbcL* (rubisco large subunit), and *RCA* (rubisco activase) (Figures 6C–F). It is rational to propose that *ZjPPH* could decrease the photosynthetic efficiency, which should be further evidenced by the determination of ultrastructure characteristics of chloroplasts and the detailed photosynthetic parameters.

### Overexpression of *ZjPPH* Caused Ultrastructure Changes of Chloroplasts

**FIGURE 7:**
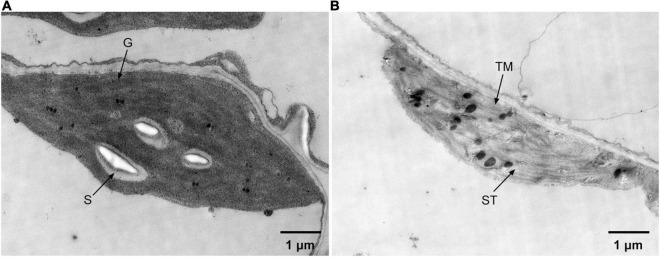
Ultrastructure of chloroplasts in panel **(A)**. Control **(B)**. *ZjPPH*-overexpressing plants. ST, stroma; S. starch; G. grana stacks; TM, thylakoid membrane.

Transmission electron microscopic analysis was carried out to reveal the ultrastructure changes of chloroplasts caused by the overexpression of *ZjPPH*. The results revealed that the overexpression of *ZjPPH* caused drastic changes to the ultrastructure of chloroplasts. In detail, the chloroplasts in the control maintained a high stability status with normal grana stacks, starch particles, and thylakoid membrane (Figure 7A). In contrast, the chloroplasts showed a decomposition tendency in the *ZjPPH*-overexpressing plants with fewer grana stacks and looser thylakoid membrane (Figure 7B).

### The JIP Test Revealed That the Overexpression of *ZjPPH* Suppressed Photosynthetic Efficiency

**FIGURE 8:**
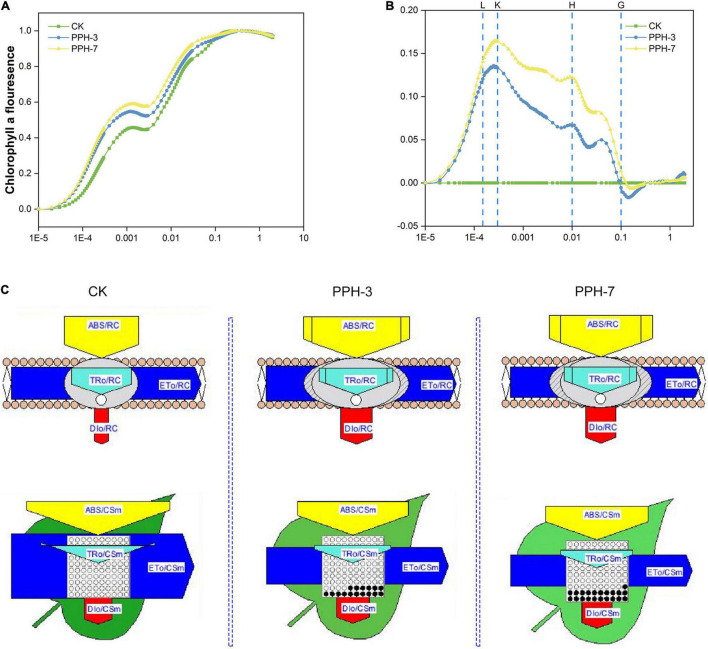
Prompt chlorophyll *a* fluorescence transient analysis of the *ZjPPH*-overexpressing lines. **(A)** The standard OJIP curves. **(B)** The relative variable fluorescence V_*t*_ of the transient chlorophyll *a* fluorescence. **(C)** The proportion of phenomenological energy flux parameters of the transgenic lines and the control displayed by the leaf pipeline model. The fluorescence parameters were displayed as means of ten replicates. The L, K, H, and G bands between the transgenic lines and control were significantly different at *p* ≤ 0.05 based on Student’s *t*-test. The width of the arrow represents the relative values of the associated parameters; dark circle represents non-active reaction centers.

The JIP test revealed that F_*O*_/F_*V*_ and F_*V*_/F_*M*_ were higher in the control than in the transgenic lines, indicating less of active photosynthesizing structures and photosynthetic membranes in the transgenic lines (Supplementary Table 1). Comparison of normalized OJIP curves provides detailed information of structural and functional differences of the photosystem (Hu et al., 2016). As shown in Figure 8A, the O-J step values were higher in the transgenic lines than in the control. It demonstrated that the expression of *ZjPPH* retarded the electron flow in the acceptor side of PSII.

The L-band is an indicator of the PSII status in the thylakoid grana membrane. A positive L-band was observed, suggesting the ungrouping of the antenna complexes and attenuated connectivity between LHCII and PSII reaction centers (Figure 8B and Supplementary Figure 1A). The positive K-band revealed the inactivation of OEC, resulting in the slower electron transport toward PSII reaction center (Supplementary Figure 1B). In this study, no obvious H-band was identified, indicating that the expression of *ZjPPH* caused no significant changes of the PQ pool volume and electron transduction between the two PS (Supplementary Figure 1C). The negative G-band implied that the PSI acceptors pool is bigger (Supplementary Figure 1D). Because the intersystem electron transduction did not show significant difference, it is rational to conclude that the PSI was suppressed in the transgenic lines. The lower RE_0_/CS_*m*_ of the transgenic lines also supported this assumption (Supplementary Table 1).

Phenomenological energy pipeline models help to visualize the influence caused by the overexpression of *ZjPPH*. It showed that membrane model parameters, including ABS/RC, TR_0_/RC, and DI_0_/RC, were increased in the transgenic lines (Figure 8C). However, the ET_0_/RC was showed no significant changes. It indicated the decreased efficiency of PSII reaction center in electron transport (Janeeshma et al., 2021). Cross-section (CS)-level indicators, including ABS/CS_*m*_, TR_0_/CS_*m*_, and ET_0_/CS_*m*_, were lower in the transgenic lines, while the DI_0_/CS_*m*_ increased (Figure 8C). In addition, less active reaction centers were identified in the *ZjPPH*-overexpressing lines. The decrease of ABS/CS_*m*_, TR_0_/CS_*m*_, and ET_0_/CS_*m*_ was related to the increased inactive reaction centers and suppressed PSII efficiency (Janeeshma et al., 2021). The increase of the DI_0_/CS_*m*_ reflected the inefficiency in the utilization of light energy.

## Discussion

Interpreting the chlorophyll degradation and photosynthetic mechanism is of vital importance for prolonging the green period of *Z. japonica*. In this study, the *PPH* gene was successfully isolated, and bioinformatic analysis proved that it is a member of α/β hydrolases. The conserved PPH motif with a Ser residue confirmed its role as a serine-type hydrolase to PPH belongs (Guyer et al., 2018). The indicator of gene function prediction, Ka/Ks ratio (Gaut et al., 1996), demonstrated that ZjPPH is functionally conserved. Transient expression analysis demonstrated that ZjPPH was a chloroplast-specific localized protein, and it is in consistent with the homologous proteins in Arabidopsis (Schelbert et al., 2009), bamboo (Wei et al., 2013), and perennial ryegrass (Zhang et al., 2016).

The promoter activity characterization and gene transcriptional expression analysis parallelly revealed that *ZjPPH* was expressed in both leaves and roots and could be induced by ABA and dark treatments. The expression of rice *PPH* was detected in the root, supporting our assumption that *ZjPPH* might be involved in other activities besides photosynthesis (Morita et al., 2009). *LpPPH* was reported to be upregulated by exogenous ABA, and the expression of bamboo *PPH* increased after darkness. As revealed by previous works, the expression of *PPH* increased to a rather high level during senescence (Schelbert et al., 2009; Zhang et al., 2016). In this study, *ZjPPH* expression level is positively associated with senescence, suggesting its role in senescence process. MeJA and SA were important hormones actively involved in senescence (Schippers et al., 2015). Different from the promoter activity analysis, the qRT-PCR results revealed that *ZjPPH* could also be upregulated by MeJA and SA, indicating the role of *ZjPPH* in MeJA and SA-induced senescence process. The different sampling time might be responsible for this result. In general, promoter activity characterization with the GUS reporting system usually needs longer incubating time than gene expression-level test. *PPH* is involved in chlorophyll degradation during leaf senescence in Arabidopsis (Schelbert et al., 2009). The heterologous expression of bamboo *PPH* in Arabidopsis caused fast yellowing phenotype (Wei et al., 2013). Transient expression of *LpPPH* caused chlorophyll breakdown in *N. benthamiana* (Zhang et al., 2016). In agreement with the previous reports, the functionality of *ZjPPH* was confirmed in accelerating chlorophyll degradation in this study. The ABA and soluble sugar contents determination revealed the expression of *ZjPPH* led to the accumulation of ABA and soluble sugar. ABA and sugar accumulation play positive roles in promoting senescence (Wingler et al., 2005; Schippers et al., 2015). *SAG12* and *SAG14* were senescence markers (Kim et al., 2008; Schelbert et al., 2009). The transcript level of *SAG12* and *SAG14* was increased, indicating that the senescence processes were activated in the transgenic lines. It was in accordance with the findings in perennial ryegrass (Zhang et al., 2016). The transcript abundance of the photosynthetic markers, such as *CAB1*, *PsaF*, *RbcL*, and *RCA*, was decreased, leaving a hint of photosynthesis retardation in the transgenic lines (Sun et al., 2016). The transcript abundance of the photosynthetic marker was decreased, leaving a hint of photosynthesis retardation in the transgenic lines.

Arabidopsis *pph-1* mutant showed nearly unchanged thylakoid stacks and unreduced membrane density compared with wild type after dark-induced senescence (Schelbert et al., 2009). The same is true of rice *pph* mutant in which the decomposition of the chloroplast ultrastructure was retarded during dark incubation (Morita et al., 2009). Our TEM observation revealed that the overexpression of *ZjPPH* promoted the decomposition of photosynthesis system, proving the important roles of *ZjPPH* in adjusting photosynthetic systems of chloroplast. JIP test results supported the TEM observation that the antenna complexes were ungrouped and the connectivity between LHCII and PSII reaction centers was attenuated. It has been reported that PPH accelerates PSI reaction center and dimeric PSII protein degradation (Morita et al., 2009; Ren et al., 2010). Similar results have been obtained for the JIP test determination in this study that *ZjPPH* suppressed the efficiency both PSII and PSI. PPH was responsible for catalyzing pheophytin *a*, the primary electron acceptor in PSII. Surprisingly, the intersystem electron transport chain between PSII and PSI showed no significant differences in the transgenic lines. The decreased efficiency of energy transformation of PSII reaction center as well as the inefficiency of light energy utilization was responsible for the insensitivity of the intersystem electron transduction in the transgenic lines. It seems reasonable to speculate that *ZjPPH* suppressed photosynthesis efficiency by mainly retarding PSII and PSI, without significantly altering the intersystem electron transduction.

## Conclusion

**FIGURE 9:**
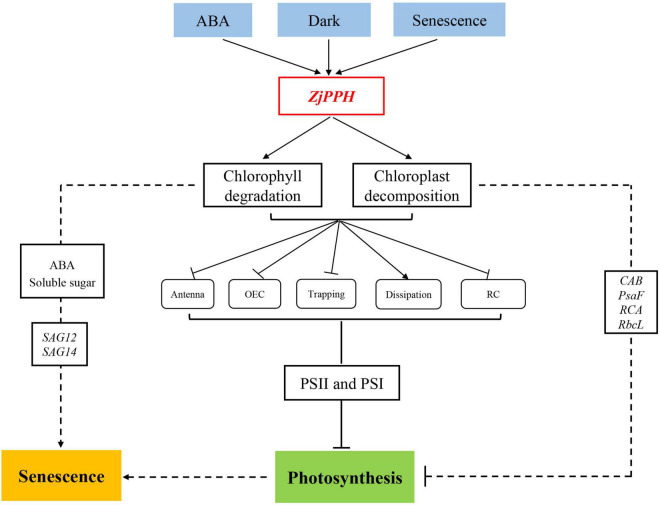
Proposed model of the molecular regulating machinery of *ZjPPH* in senescence and photosynthesis.

In summary, the proposed working model of the molecular regulating machinery of *ZjPPH* in regulating senescence and photosynthesis is summarized in Figure 9. The expression of *ZjPPH* could be induced by ABA, dark, and senescence. ZjPPH was localized in the chloroplasts and its overexpression accelerated chlorophyll degradation. *ZjPPH* accelerated senescence with the accumulation of ABA and soluble sugar contents, as well as the increased transcriptional level of *SAG12* and *SAG14*. JIP test analysis revealed that *ZjPPH* suppressed photosynthesis efficiency by mainly suppressing both PSII and PSI, and photosynthesis-related genes. Consequently, *ZjPPH* plays important roles in chlorophyll degradation and photosynthesis. It could be a valuable gene for genetic editing to cultivate new cultivars with stay-green trait and improved photosynthetic efficiency.

## Data Availability Statement

The datasets presented in this study can be found in online repositories. The names of the repository/repositories and accession number(s) can be found in the article/Supplementary Material.

## Author Contributions

XF and JW designed the study. KT, CH, and YY performed the experiments. KT, HZ, HL, and LX wrote the manuscript. All authors have read and approved the manuscript.

## Conflict of Interest

The authors declare that the research was conducted in the absence of any commercial or financial relationships that could be construed as a potential conflict of interest.

## Publisher’s Note

All claims expressed in this article are solely those of the authors and do not necessarily represent those of their affiliated organizations, or those of the publisher, the editors and the reviewers. Any product that may be evaluated in this article, or claim that may be made by its manufacturer, is not guaranteed or endorsed by the publisher.

## Footnotes

1
https://web.expasy.org/compute_pi/

2
http://www.cbs.dtu.dk/services/TargetP-1.1/index.php

## Supplementary Material

Supplementary Figure 1 Different normalizations of the fluorescence rise kinetics OJIP curves. **(A)** L-band. **(B)** K-band. **(C)** H-band. **(D)** G-band.
