# Supplementary figures and images for: Functional Characterization of the Pheophytinase Gene, ZjPPH, From Zoysia japonica in Regulating Chlorophyll Degradation and Photosynthesis
Source: Front Plant Sci. 2021 Dec 23;12:786570. doi: 10.3389/fpls.2021.786570 (PMC8733386; doi:10.3389/fpls.2021.786570)

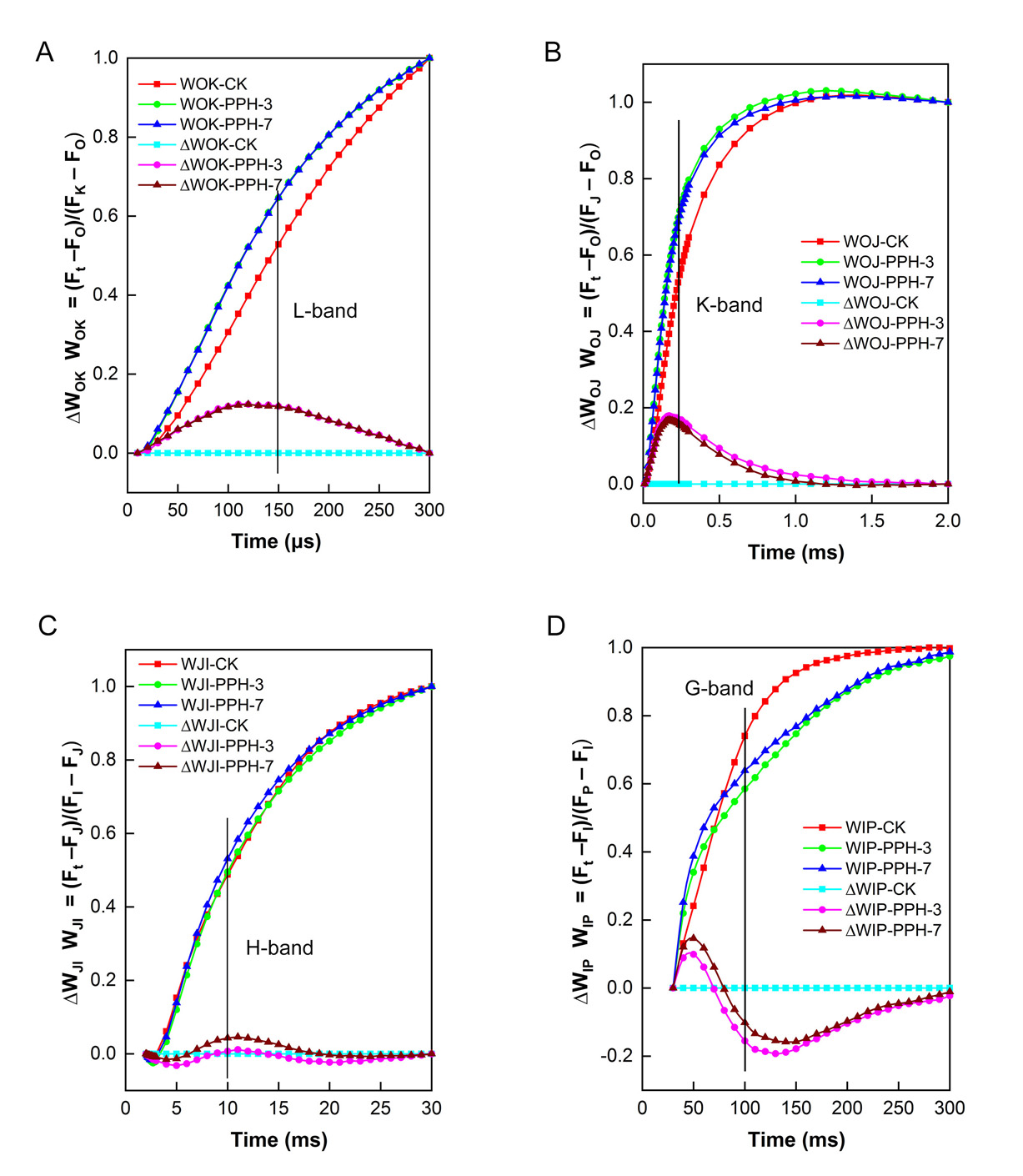

Supplement: Supplementary Figure 1 — Different normalizations of the fluorescence rise kinetics OJIP curves. (A) L-band. (B) K-band. (C) H-band. (D) G-band. [file Image_1.JPEG]
