# Supplementary material for: Functional Characterization of the Pheophytinase Gene, ZjPPH, From Zoysia japonica in Regulating Chlorophyll Degradation and Photosynthesis
Source: Front Plant Sci. 2021 Dec 23;12:786570. doi: 10.3389/fpls.2021.786570 (PMC8733386; doi:10.3389/fpls.2021.786570)
Supplement: Supplementary file 2 [file Table_1.DOCX]

Table S1 Fluorescence parameters of transgenic lines and control

| Plant | F_O_/F_M_ | F_V_/F_M_ | ψ(E_0_) | φ(E_0_) | δ(R_0_) | φ(R_0_) | RE_0_/CS_m_ | PI _ABS_ | PI _total_ | DF _Total_ |
| --- | --- | --- | --- | --- | --- | --- | --- | --- | --- | --- |
| CK | 0.14±0.005^b^ | 0.86±0.004^a^ | 0.53±0.01^a^ | 0.46±0.01^a^ | 0.29±0.04^a^ | 0.13±0.02^a^ | 481±72.3^a^ | 2.6±0.4^a^ | 1.04±0.4^a^ | 0.019±0.1^a^ |
| PPH-3 | 0.23±0.08^a^ | 0.77±0.08^b^ | 0.45±0.07^b^ | 0.35±0.08^b^ | 0.24±0.04^b^ | 0.084±0.03^b^ | 240±119.1^b^ | 0.82±0.6^b^ | 0.26±0.2^b^ | -0.59±0.5 ^b^ |
| PPH-7 | 0.21±0.06^a^ | 0.79±0.06^b^ | 0.40±0.05^b^ | 0.32±0.05^b^ | 0.18±0.03^b^ | 0.058±0.01^b^ | 155±38.4^b^ | 0.76±0.5^b^ | 0.17±0.1^b^ | -0.77±0.3^b^ |

Note: values are expressed as mean ± SD and the data is an average of 10 measurements. Different letters indicate significant differences at p ≤ 0.05 based on Fisher’s protected least significant difference (LSD) test.
